# From starting mechanical ventilation to ventilator-associated pneumonia, choosing the right moment to start antibiotic treatment

**DOI:** 10.1186/s13054-016-1342-1

**Published:** 2016-06-03

**Authors:** Paula Ramirez, Cristina Lopez-Ferraz, Monica Gordon, Alexandra Gimeno, Esther Villarreal, Jesús Ruiz, Rosario Menendez, Antoni Torres

**Affiliations:** Department of Intensive Care Medicine, Hospital Universitari i Politècnic la Fe, Valencia, Spain; Centro de Investigación Biomedica en Red-Enfermedades Respiratorias (CibeRes, CB06/06/0028), Instituto de Salud Carlos III, Madrid, Spain; Department of Pneumology, Hospital Universitari i Politècnic la Fe, Valencia, Spain; Department of Pneumology, Hospital Clinic, Barcelona, Spain

**Keywords:** Ventilator-associated pneumonia, Ventilator-associated tracheobronchitis, Inflammatory biomarker, Appropriate treatment

## Abstract

**Background:**

Ventilator-associated pneumonia (VAP) can have a clear onset or may be a result of the gradual appearance of symptoms and signs of VAP (gradual VAP). The aim of this paper is to describe the VAP development process with the intention of discriminating between those pneumonias with a clear beginning and those that are diagnosed after a period of maturation. In addition, we evaluate the effect of the starting time of antibiotic treatment in both situations.

**Methods:**

Consecutive ventilated patients fulfilling VAP criteria were included. The patients were monitored for clinical, microbiological, and inflammatory signs. Patients with VAP were classified into two groups: (1) nongradual VAP (patients in whom all VAP criteria were detected for the first time on the day of diagnosis) and (2) gradual VAP (progressive appearance of signs and symptoms throughout the pre-VAP period [<96 h to >24 h before VAP diagnosis]).

**Results:**

A total of 71 patients with VAP were identified, of whom 43 (61 %) had gradual VAP, most of whom (*n* = 38, 88 %) had late-onset VAP. Antibiotic treatment was given to 34 (79 %) patients with gradual VAP in the pre-VAP period, and empirical antibiotic treatment was appropriate in 22 patients (51 %). The patients with an appropriate empirical treatment had a higher percentage of early clinical response to treatment (68 % [*n* = 15] vs. 28 % [*n* = 7]; *p* = 0.009). An attempt was made to find a diagnostic test capable of identifying the infectious process underway, but clinical scales and biomarkers of inflammation helped us to achieve acceptable results.

**Conclusions:**

Gradual emergence of VAP, mainly of late onset, is a common condition. Clinicians should be aware of this gradual onset of the infection to establish an early antibiotic treatment, even before the classic diagnostic criteria for VAP are applied.

## Background

The diagnosis of ventilator-associated pneumonia (VAP) requires compliance with clinical, microbiological, and radiological criteria [1–3]. However, any clinician would be able to report those cases in which, although there are suggestive elements of lung infection, the criteria for a standard VAP diagnosis are not met. In these cases, doctors face the question whether to start an antibiotic treatment. This situation has probably led to the emergence of new diagnostic standards. One of these new approaches has been named *ventilator-associated tracheobronchitis* (VAT) [4–9]. In fact, lower mortality has been prospectively demonstrated in those patients with VAT appropriately treated with antibiotics [9]. However VAT differs from VAP only by the absence of pulmonary infiltrates on chest x-rays, and pulmonary consolidation can frequently be misdiagnosed on the basis of chest x-rays of mechanically ventilated patients [2, 10].

According to clinical practice, it appears that the progressive development of VAP requires a more complex approach than the radiological differentiation between VAT and VAP. From the start of mechanical ventilation, artificial airway and bacterial colonization pose a challenge to the patient´s host defense system. As a result of this challenge, patients can present with fever, an elevated white cell count, or an increase in the amount of respiratory secretions (and even the development of purulence). A respiratory culture will reveal the presence of microorganisms. Throughout this confrontation, clinicians should recognize the right moment to start antibiotic treatment.

We hypothesized the existence of two different pathways to VAP: VAP with a clear onset and VAP arising after a prodromal development period. In this paper, we aim to describe both pathways analyze the effect of starting antibiotic treatment in the early stages.

## Methods

### Study design and inclusion criteria

We performed a prospective observational study in a 24-bed medical intensive care unit (ICU) of a 1200-bed university hospital over an 18-month period. All patients under mechanical ventilation (for at least 48 h) were followed for the development of VAP. Nonquantitative tracheobronchial aspirates (TBAS) and serum inflammatory biomarkers were analyzed every 48–72 h. Patients with VAP were included in the study and were classified into two groups: (1) gradual VAP and (2) nongradual VAP. The Hospital la Fe Institutional Review Board approved the study, and informed consent was obtained from the patients’ relatives.

### Data collection protocol

The following data were collected upon enrollment into the study: sex, comorbidities, and severity scores before intubation (including Acute Physiology and Chronic Health Evaluation II score) [11], Sepsis-related Organ Failure Assessment score [12], and modified Clinical Pulmonary Infection Score [mCPIS] [13]), together with the reason for ICU admission and for starting mechanical ventilation. Pulmonary infection was monitored by means of daily evaluation of signs and symptoms and by calculating the mCPIS. The presence of nonpulmonary infections was established according to Centers for Disease Control and Prevention criteria [14].

### Definitions

#### VAP

VAP was defined as two or more of the following: temperature above 38 °C, white cell count above 12,000/mm^3^ or below 4000/mm^3^, or purulent respiratory secretions, plus a new or progressive pulmonary infiltrate on the chest x-ray. VAP confirmation was defined by the quantitative culture of TBAS greater than or equal to 10^5^ cfu/ml^2^, bronchoalveolar lavage (BAL) with at least 10^4^ cfu/ml, or mini-BAL with at least 10^3^ cfu/ml [1–3, 15].

#### Gradual VAP

Gradual VAP was defined as presence in the pre-VAP period of purulent respiratory secretions, plus one or both of the following: temperature above 38 °C and a white cell count greater than 12,000/mm^3^, and without a new or progressive pulmonary infiltrate on a chest x-ray.

#### Nongradual VAP

Nongradual VAP was defined as VAP not fulfilling gradual VAP criteria.

#### Pre-VAP period

**Fig. 1:**
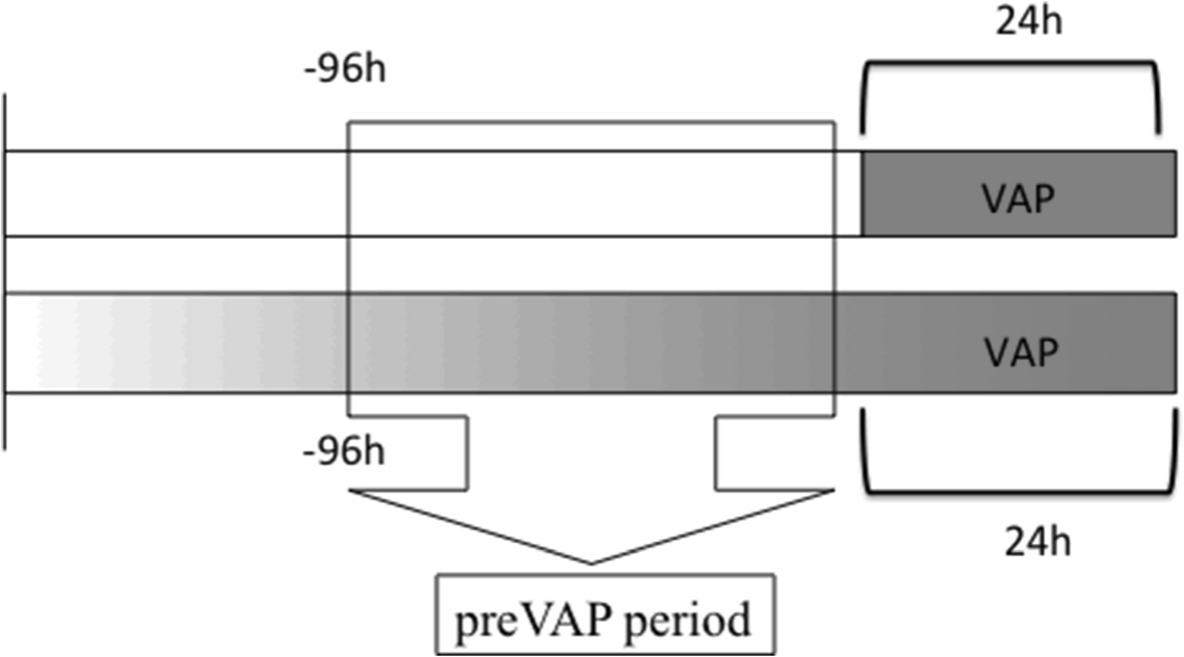
Time frames in the gradual and nongradual ventilator-associated pneumonia (VAP) groups. Colour represents an abrupt VAP onset in the first line and a gradual VAP onset in the second

The pre-VAP period was defined as less than 96 h and more than 24 h before VAP diagnosis. The pre-VAP period is shown in Fig. 1.

### Assessment of VAP clinical response at 72 h

Nonresponders were considered as those patients in whom at least one of the following criteria was present: (1) absence of improvement in the ratio of partial pressure of arterial oxygen to fraction of inspired oxygen; (2) persistence of fever (≥38 °C), or hypothermia (<35 °C), together with purulent respiratory secretions; (3) greater than 50 % increase in respiratory infiltrate on a chest x-ray; and (4) the development of septic shock or multiorgan failure [16].

### Study of inflammatory markers

Blood samples were centrifuged (1500 rpm for 10 minutes), and the separated serum was frozen at −80 °C. Procalcitonin (PCT) was measured using time-resolved amplification of cryptate emission technology in a KRYPTOR analyzer (B∙R∙A∙H∙M∙S, Berlin, Germany). C-reactive protein (CRP) was measured using an immunoturbidimetric method with a commercially available kit (Tina-quant C-Reactive Protein; Roche Diagnostics, Mannheim, Germany).

### Statistical analysis

Continuous variables were compared using Student’s *t* test for normally distributed variables and the Mann-Whitney *U* test for nonnormally distributed variables. Categorical variables were compared using the χ^2^ and Fisher’s exact tests, where appropriate. The threshold for statistical significance of the tests was set at 5 %. Collected data were entered and analyzed using SPSS 15.0 software (SPSS, Chicago, IL, USA).

## Results

### Description of the population

**Fig. 2:**
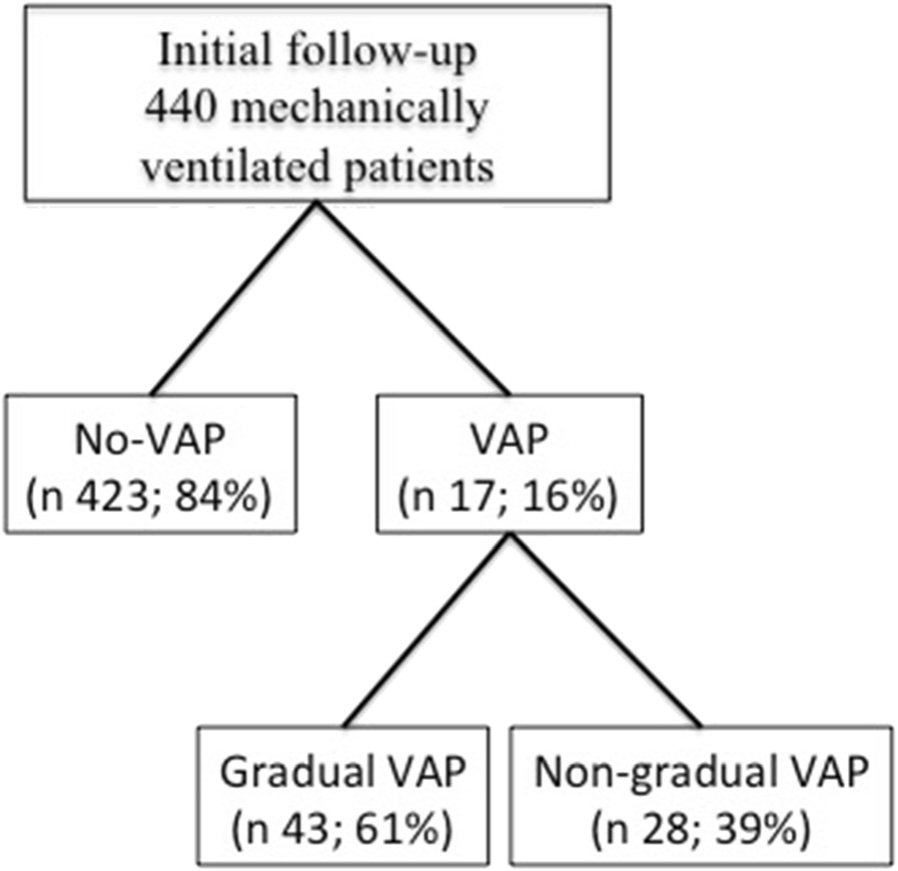
Inclusion algorithm. *VAP* ventilator-associated pneumonia

Four hundred forty patients receiving mechanical ventilation were screened during the study period, and 71 (16 %) patients with VAP were identified. Of these 71 patients, 43 (61 %) had gradual VAP and 28 (39 %) had nongradual VAP (Fig. 2). The reasons for ICU admission and patient comorbidities are summarized in Table 1. Patient characteristics at ICU admission were similar between the two groups.

**Table 1:** Patient characteristics at intensive care unit admission

	All patients (*n* = 71)	Nongradual VAP (*n* = 28)	Gradual VAP (*n* = 43)	*p* Value
Age, years	61 [53–71.5]	60 [54.5–73]	63 [50–71]	0.874
Male sex	45 (63 %)	15 (53 %)	30 (70 %)	0.166
Smoking	29 (41 %)	9 (32 %)	20 (46 %)	0.229
Alcohol	14 (20 %)	9 (32 %)	5 (12 %)	0.034
APACHE II score	23 [18.5–28.5]	21.5 [18.5–25.5]	25 [18.5–31.5]	0.166
Comorbidities				
Hypertension	32 (45 %)	12 (43 %)	20 (46 %)	0.762
Diabetes mellitus	17 (24 %)	8 (28 %)	9 (21 %)	0.461
Chronic cardiac failure	3 (4 %)	0	3 (7 %)	0.171
Chronic renal failure	8 (11 %)	2 (7 %)	6 (14 %)	0.466
Chronic lung disease/COPD	11 (15 %)	3 (11 %)	8 (19 %)	0.469
Immunosuppression	22 (31 %)	8 (29 %)	14 (32 %)	0.723
Reason for intubation				
Respiratory failure	29 (41 %)	11 (39 %)	18 (42 %)	0.754
Cardiovascular failure	12 (17 %)	5 (18 %)	7 (16 %)	0.488
Coma	30 (42 %)	12 (43 %)	18 (42 %)	0.813
SOFA	11 [7.5–12.5]	11 [7–13]	11 [8–12]	0.928
CPIS	4 [3.5–5]	4 [3.5–5]	4 [3–5]	0.431
PaO_2_/FiO_2_	195 [100–250]	175 [120.5–250]	200 [69.5–250]	0.816
Temperature, °C	36.3 [36–36.9]	36.2 [36–36.9]	36.5 [36–36.9]	0.815
Leukocytes, cells/mm^3^	11,100 [9150–14,900]	11,100 [10600–12,300]	11,750 [8500–20,800]	0.729
CRP, mg/L	28 [0.5–104]	111 [6–250]	19 [0.5–70.2]	0.165
PCT, ng/ml	0.26 [0.01–1.44]	0.39 [0.01–1.44]	0.17 [0.01–0.76]	0.743

*APACHE II* Acute Physiology and Chronic Health Evaluation II, *COPD* chronic obstructive pulmonary disease, *SOFA* sepsis organ failure score, *CPIS* Clinical Pulmonary Infection Score, *CRP* C-reactive protein, *PCT* procalcitonin, *PaO*_*2*_ */FiO*_*2*_ ratio of partial pressure of arterial oxygen to fraction of inspired oxygen

Results are expressed as median [interquartile range] for continuous variables and number (%) for categorical variables

### Pre-VAP period characteristics

Table 2 shows the characteristics of the patients in the pre-VAP period. Patients with gradual VAP had a higher CPIS in the pre-VAP period (5 [5–7] vs. 4 [1.5–5]; *p* = 0.018). Although not statistically significant, serum CRP was clearly higher in patients with gradual VAP. Airway colonization was more frequent in patients with gradual VAP; the microorganisms involved were *Acinetobacter baumannii* (33 %), *Pseudomonas aeruginosa* (18 %), and methicillin-sensitive *Staphylococcus aureus* (9 %). Most (79 %) of the patients with gradual VAP received antibiotic treatment in the pre-VAP period due to the presence of lung infection symptoms. The antibiotic treatment was appropriate in 51 % of cases (*n* = 22). None of the patients with nongradual VAP were treated in the pre-VAP period.

**Table 2:** Patient characteristics in pre-VAP period

	All patients (*n* 71)	Nongradual VAP (*n* 28)	Gradual VAP (*n* 43)	*p* Value
Airway bacterial colonization	62 (87 %)	19 (68 %)	43 (100 %)	<0.001
SOFA	7.5 [5–10.5]	8 [0–10]	7 [5.5–10.5]	0.922
mCPIS	5 [4–6]	4 [1.5–5]	5 [5–7]	0.018
PaO_2_/FiO_2_	200 [148–274]	200 [140.5–267]	200 [164–280]	0.596
Temperature, °C	37 [36.2–38]	36.4 [36–37.9]	37.2 [36.6–38]	0.05
Leukocytes, cells/mm^3^	12,400 [7900–16,800]	11,550 [8200–17,500]	12,700 [7200–16,550]	0.984
CRP, mg/L	134 [52–208]	76 [13.5–164]	159 [80–210]	0.051
PCT, ng/ml	0.83 [0.37–3.96]	0.78 [0.27–7.6]	0.9 [0.43–2.16]	0.953

*SOFA* sepsis organ failure score, *mCPIS* modified Clinical Pulmonary Infection Score, *CRP* C-reactive protein, *PCT* procalcitonin, *PaO*_*2*_ */FiO*_*2*_ ratio of partial pressure of arterial oxygen to fraction of inspired oxygen, *VAP* ventilator-associated pneumonia

Results are expressed as median [interquartile range] for continuous variables and number (%) for categorical variables

### VAP characteristics

Table 3 shows the characteristics of patients at the time of VAP diagnosis (gradual vs. nongradual VAP). No differences could be found with regard to etiology, inflammatory response, severity scores, or outcomes depending on the presence (or not) of a developing period. Late-onset VAP was more common in the gradual VAP group (38 [88 %] vs. 12 [42 %]; *p* < 0.001). The VAP origin was due to the microorganisms isolated at the time of the developing period in 100 % of cases.

**Table 3:** Patient characteristics at time of ventilator-associated pneumonia diagnosis

	All patients (*n* = 71)	Nongradual VAP (*n* = 28)	Gradual VAP (*n* = 43)	*p* Value
MV duration before VAP, days	7 [4.5–10.5]	4.5 [3–9]	8 [6.5–11.5]	0.002
Early VAP	21 (29 %)	16 (57 %)	5 (12 %)	<0.001
SOFA score	5.5 [8–11.5]	10 [8–12]	7 [5–11]	0.142
mCPIS score	7 [6–8]	7 [6–8]	7 [6–8]	0.908
PaO_2_/FiO_2_	197 [137–247]	200 [160–282]	180 [130–226]	0.119
Temperature, °C	38 [37–38.7]	38 [36.7–38.6]	38 [37–38.7]	0.928
Leukocytes, cells/mm^3^	12250 [8050–17,400]	12,000 [9100–16,800]	12,400 [7500–17,500]	0.930
CRP (mg/L)	151 [90–315]	200 [100–340]	132 [73.5–265]	0.301
PCT (ng/ml)	1.4 [0.5–4.3]	1.4 [0.5–6.08]	1.4 [0.5–4.1]	0.961
Etiology				
Nonfermenting GNB	42 (52 %)	17 (61 %)	25 (58 %)	0.829
*Enterobacteriaceae*	14 (20 %)	6 (21 %)	8 (19 %)	0.770
*Staphylococcus aureus*	8 (11 %)	2 (7 %)	6 (14 %)	0.315
*Haemophilus influenzae*	5 (7 %)	2 (7 %)	3 (7 %)	0.661
*Streptococcus*	1 (1 %)	0	1 (2 %)	0.606
*Aspergillus*	1 (1 %)	1 (3 %)	0	0.394
Treatment failure (72 h)	39 (55 %)	17 (61 %)	22 (51 %)	0.442
Microbial persistence (72 h)	43 (61 %)	16 (57 %)	27 (63 %)	0.361
Relapse	8 (11 %)	3 (11 %)	5 (11 %)	0.121
ICU stay, days,)	19 [13–29]	16 [12–28.5]	20.5 [14–29]	0.346
Hospital stay, days	24 [12–51]	19.5 [9–42]	29 [16–57]	0.054
Days of MV	13 [8–18]	13.5 [8.5–19.5]	12.5 [8–17]	0.679
28-day mortality	43 (61 %)	18 (64 %)	25 (58 %)	0.605

*MV* mechanical ventilation, *VAP* ventilator-associated pneumonia, *SOFA* Sepsis-related Organ Failure Assessment, *mCPIS* modified Clinical Pulmonary Infection Score, *CRP* C-reactive protein, *PCT* procalcitonin, *GNB* gram-negative bacteria, *PaO*_*2*_ */FiO*_*2*_ ratio of partial pressure of arterial oxygen to fraction of inspired oxygen, *ICU* intensive care unit

Results are expressed as median [interquartile range] for continuous variables and number (%) for categorical variables

Appropriate antibiotic treatment introduced in the pre-VAP period in the gradual VAP group was associated with a higher rate of early clinical response (68 % [*n* = 15] vs. 28 % [*n* = 7]; *p* = 0.009). However, ICU mortality and 28-day mortality were not influenced by the use of appropriate treatment in the pre-VAP period.

### Biomarker kinetics

CRP and PCT kinetics from the pre-VAP period to the diagnosis of the infection were analyzed and compared between the two groups (Table 4). In both groups, biomarkers showed an increasing pattern (statistically significant for PCT), except for CRP in gradual VAP.

**Table 4:** Biomarker kinetics

	Pre-VAP CRP (mg/dl)	VAP CRP (mg/ml)	*p* Value	Pre-VAP PCT (ng/dl)	VAP PCT (ng/ml)	*p* Value
Nongradual VAP	76 [9.75–186]	200 [99–347]	0.565	0.78 [0.25–9.09]	1.41 [0.5–6.2]	0.006
Gradual VAP	159 [80–210]	132 [67.7–268.5]	0.502	0.9 [0.42–2.35]	1.42 [0.56–4.21]	0.008

*CRP* C-reactive protein, *PCT* procalcitonin, *VAP* ventilator-associated pneumonia

### Antibiotic treatment guide

Recognized cutoffs for antibiotic guidance in serum PCT (≥0.5 ng/ml and >1 ng/ml) and modified mCPIS (>5 points) were assessed to establish their capacity to identify an ongoing infection. Better rates were achieved with mCPIS (sensitivity 44 %, specificity 92 %, positive predictive value 92 %, negative predictive value 48 %, (Likelihood ratio) LH+ 6.67, LH− 0.60, AUC 0.670, 95 % CI 0.545–0.796; *p* = 0.016). The AUCs for CRP and PCT were 0.678 and 0.505, respectively. CRP and PCT were ineffective in identifying gradual VAP (Table 5).

**Table 5:** Identification of gradual ventilator-associated pneumonia with assessment of diagnostic tool parameters

	AUC	95 % CI	*p* Value	Sensitivity	Specificity	Likelihood ratio	
						Positive	Negative
PCT ≥0.5 ng/ml	0.505	0.332–0.677	0.953	65	40	1.08	0.88
CRP ≥54 mg/dl	0.678	0.493–0.863	0.047	60	49	1.22	0.86
mCPIS >5 points	0.670	0.545–0.796	0.016	44	92	6.67	0.60

*CRP* C-reactive protein, *mCPIS* modified Clinical Pulmonary Infection Score, *PCT* procalcitonin

## Discussion

To our knowledge, we are the first to describe the concept of gradual VAP. Establishment of gradual VAP is a common process, mainly in the case of late pneumonia. More importantly, our data support the need to initiate proper antibiotic treatment in the early stages of infection without waiting for all the diagnostic criteria for VAP to be met. However, in this study, we were unable to identify effective diagnostic tools for gradual VAP, apart from the usual clinical criteria.

In line with the idea proposed by Craven and Hjalmarson [4], our study supports the hypothesis of a continuum between airway colonization, an intermediate process (called *ventilator-associated tracheobronchitis* in their study), and VAP. During this developing period, the patient’s immune system will attempt to prevent the spread of the organism, and signs of this confrontation will be observable in the form of fever, leukocytosis, and purulent bronchial secretions. This process is typical in late-onset VAP (76 % of late VAP cases in our series were gradual VAP), and its absence is probably justified by the presence of a sudden and large bacterial inoculum into the lung (inoculate at intubation in early VAP, and due to accidental aspiration of subglottic secretions in late VAP).

Our results do not support a nosological separation between VAP and VAT. As described in the literature, VAP and VAT are distinguishable only by the presence of visible lung infiltrate on a chest x-ray. However, the discriminative ability of a portable chest x-ray is more than doubtful. In fact, even in community-acquired pneumonia, plain radiography has shown a diagnostic inaccuracy of 17 % compared with chest computed tomography [10]. Nonetheless, the value of studying VAP development lies in antibiotic prescription rather than in a diagnostic disquisition.

In our study, appropriate antibiotic treatment introduced in the developing period in gradual VAP was associated with a higher rate of early clinical response. Therefore, identifying the right moment to start antibiotics in this continuum between colonization and VAP seems to be a challenge. PCT may be a valuable tool in this context. Stolz et al. showed a safety algorithm for antibiotic discontinuation based on serum PCT after 72 h of antibiotic treatment in 101 patients with VAP [17]. In our study, gradual VAP showed serum PCT greater than or equal to 0.5 ng/ml during the developing period in 65 % of cases, but this was also true for nongradual VAP in 60 % of cases.

According to our results, in the event of suspected VAP, a mCPIS score higher than 5 could indicate antibiotic initiation. However, the lack of sensitivity of mCPIS in our study precludes a safe use of this tool to rule out the existence of an ongoing infectious process. Using a different methodological approach, Singh et al. demonstrated the usefulness of CPIS to safely withdraw antibiotics after 72 h of treatment in patients with an initial but incomplete diagnosis of VAP (CPIS ≤6 points at inclusion) [18].

Our study has several limitations. The sample size is not large and could be responsible for the observed negative results. However, our sample size is not too far removed from that used in studies by Stolz et al. [17] and Singh et al. [18]. Gradual VAP definition is roughly equal to VAT criteria, but in our case it systematically preceded the diagnosis of VAP. In any case, we avoid discussion of labeling of patients, as our objective is to analyze the correct time for starting antibiotic treatment. In this sense, the progression to VAP is marked, despite an appropriate antibiotic treatment. Although this phenomenon has already been described [9], we would need to analyze the characteristics of patients in similar clinical conditions, but without progression to VAP, to get convincing explanations. We believe that nongradual late VAP may be due to accidental inoculation of heavily colonized oropharynx secretions. However, we had little knowledge regarding the existence of this type of incident in such cases. Finally, we must assume that demonstration of the clinical importance of the concept of gradual VAP requires additional studies based on therapeutic interventions. Since this type of intervention may lead to a greater consumption of antibiotics, it should always be associated with an antimicrobial stewardship program.

## Conclusions

Not all pneumonias are developed similarly, which is probably due to differences in the pathogenic mechanism. But what is really important is the identification of the infectious process in place, in order to start appropriate antimicrobial therapy. According to our results, antibiotic treatment should not be delayed, even if the patient does not meet the prescriptive diagnostic criteria for VAP.

## Abbreviations

APACHE II, Acute Physiology and Chronic Health Evaluation II; BAL, bronchoalveolar lavage; COPD, chronic obstructive pulmonary disease; CPIS, Clinical Pulmonary Infection Score; CRP, C-reactive protein; GNB, gram-negative bacteria; ICU, intensive care unit; mCPIS, modified Clinical Pulmonary Infection Score; MV, mechanical ventilation; PaO_2_/FiO_2_, ratio of partial pressure of arterial oxygen to fraction of inspired oxygen; PCT, procalcitonin; SOFA, Sepsis-related Organ Failure Assessment; TBAS, tracheobronchial aspirates; VAP, ventilator-associated pneumonia; VAT, ventilator-associated tracheobronchitis.
